# The relationship between perceived academic stress and college students’ employment anxiety: the mediating role of psychological resilience

**DOI:** 10.3389/fpsyt.2025.1602808

**Published:** 2025-06-03

**Authors:** Mengshan Yang, Xin Li, Xiaoye Qin, Xusheng Tian, Hao Zhang, Hongjuan Wen

**Affiliations:** ^1^ School of Economics, Zhejiang Gongshang University, Zhejiang, Hangzhou, China; ^2^ School of Statistics and Mathematics, Zhejiang Gongshang University, Zhejiang, Hangzhou, China; ^3^ Department of Student administration, the Third Affiliated Hospital of Changchun University of Traditional Chinese Medicine, Jilin, Changchun, China; ^4^ The First Clinical Medical College, Heilongjiang University of Chinese Medicine, Heilongjiang, Harbin, China; ^5^ Basic Medical College, Heilongjiang University of Chinese Medicine, Heilongjiang, Harbin, China; ^6^ College of Management, Changchun University of Traditional Chinese Medicine, Jilin, Changchun, China

**Keywords:** academic stress, employment anxiety, psychological resilience, university students, conservation of resources theory

## Abstract

**Background:**

University graduates increasingly face academic and employment-related pressures, particularly in the post-COVID-19 era. Employment anxiety has emerged as a significant mental health issue during the transition from school to work, but its underlying mechanisms remain underexplored.

**Objective:**

This study aimed to investigate the mediating role of psychological resilience in the relationship between academic stress and employment anxiety among Chinese undergraduate students.

**Methods:**

A cross-sectional survey was conducted among 1124 students from three universities in China. Participants completed validated measures of academic stress, psychological resilience, and employment anxiety. Pearson correlation analysis and mediation testing were performed using PROCESS macro Model 4, with 5000 bootstrap samples.

**Results:**

Academic stress was positively associated with employment anxiety (*B*=0.421, *p*<0.001) and negatively associated with psychological resilience (*B*=–0.230, *p*<0.001). Psychological resilience negatively predicted employment anxiety (*B*= –0.444, *p*<0.001) and partially mediated the relationship between academic stress and employment anxiety. The indirect effect accounted for 19.50% of the total effect. After controlling for gender, grade, family income, and internship experience, the mediation remained significant and robust.

**Conclusion:**

Psychological resilience plays a protective mediating role in the link between academic stress and employment anxiety. These findings underscore the importance of enhancing resilience through targeted interventions to reduce employment-related anxiety among university students. Educational institutions should integrate resilience-building strategies into academic and career counseling to promote student mental health and employability.

## 1 Introduction

### 1.1 Background

In the aftermath of the COVID-19 pandemic, university graduates have encountered an increasingly uncertain and competitive labor market, which has led to a notable rise in employment anxiety among students (1). Employment anxiety not only compromises students’ mental health but also interferes with their academic engagement and career decision-making processes. Previous research has shown that employment anxiety is associated with lower job-seeking efficacy, career indecision, and overall psychological distress (2, 3). A recent study on Chinese university graduates confirmed widespread employment anxiety, with significant gender and discipline differences (4, 5).

One key contributor to such anxiety is academic stress—defined as the psychological tension arising when academic demands exceed an individual’s coping capacity (6). Academic stress is a well-established predictor of depression (7), anxiety (8), and poor academic performance (9). Students under high academic stress are more prone to burnout, emotional exhaustion, and anticipatory worry about post-graduation transitions (10, 11). In the context of a volatile job market, academic stress may intensify concerns about employability and future career success. This concern has become particularly salient for final-year students preparing for graduation amidst economic volatility and intensified job competition (12).

Importantly, recent studies have emphasized how adverse experiences during the COVID-19 pandemic continue to influence young people’s emotional functioning and well-being, highlighting the importance of internal protective mechanisms such as resilience in buffering stress responses (13–15). Despite this, limited empirical research has investigated how academic stress translates into employment anxiety through these mechanisms.

### 1.2 Theoretical framework

To understand this process, the Conservation of Resources (COR) theory provides a compelling framework. COR theory posits that individuals strive to obtain, retain, and protect valued resources—including time, energy, social capital, and psychological assets (16). When these resources are threatened or depleted, individuals experience psychological stress, and if no adequate recovery occurs, a spiral of resource loss may ensue, increasing susceptibility to further stressors (17).

Academic stress, within this framework, represents a chronic condition of resource depletion. It taxes students’ emotional and cognitive reserves, disrupts recovery opportunities (such as rest or social engagement), and creates a perceived gap between environmental demands and internal capacities (18, 19). Over time, this mismatch can undermine students’ confidence in coping with both academic and employment-related challenges (20).

Psychological resilience, in contrast, functions as a key internal resource that facilitates adaptive recovery and emotional regulation in stressful conditions (21). Resilient individuals can draw upon stable psychological resources—such as optimism, cognitive flexibility, and emotional endurance—to preserve functioning and prevent further loss (22, 23). As such, resilience not only moderates the impact of stress but may mediate its effects on downstream psychological outcomes, such as anxiety, by interrupting the resource loss cycle (24).

Recent empirical studies support the mediating role of resilience in academic and career contexts. For example, one study found that psychological resilience mediated the relationship between academic stress and depression among medical students, suggesting that resilience functions as a protective buffer against stress-induced distress (25). Another study conducted in China indicated that resilience indirectly influenced mental health literacy and anxiety through reductions in work-related stress (26).

In the context of employment anxiety, resilience may play an especially crucial role. As students confront uncertainties about graduation, labor market volatility, and self-perceived unpreparedness, psychological resilience allows them to reinterpret challenges as manageable and to maintain forward-focused behaviors (27). This reappraisal mechanism can reduce ruminative thought patterns, buffer perceived threat, and enhance control appraisal—core psychological processes implicated in anxiety reduction (28).

Moreover, cross-cultural studies suggest that resilience-based coping may be particularly relevant in collectivist societies like China, where high expectations regarding academic and occupational success co-exist with limited emotional expression (29). In such contexts, internal resources like resilience may serve as primary coping strategies in the face of stressors that cannot easily be renegotiated or externally resolved.

Taken together, these findings suggest that psychological resilience may serve not only as a moderator that buffers stress responses but also as a mediator that transmits the effects of academic stress to psychological outcomes like employment anxiety. This mediating role is consistent with COR theory’s emphasis on dynamic resource flow and the pivotal role of internal reserves in the stress-adaptation cycle (30).

### 1.3 Research gap and hypothesis development

Although academic stress and employment anxiety have been independently linked to poor psychological outcomes, few studies have explored how resilience mediates the association between them. Prior research has focused primarily on general anxiety or depression, without accounting for the employment-specific psychological impacts faced by graduating students (31, 32). Moreover, the transitional stress of entering the workforce after the pandemic presents a distinct and underexplored challenge.

The present study aims to fill this gap by examining whether psychological resilience mediates the effect of academic stress on employment anxiety among Chinese university students. This model is particularly relevant in the post-COVID context, where resource scarcity, disrupted academic routines, and job insecurity have jointly intensified stress exposure.

Importantly, the present study also expands the literature by addressing employment anxiety as a distinct outcome—rather than general distress—highlighting the psychological risks associated with the education-to-employment transition. As previous work has shown, this period is marked by heightened vulnerability due to career uncertainty, economic instability, and intensified global competition (33).

### 1.4 Research hypotheses

Based on COR theory and the reviewed literature, the following hypotheses are proposed:

H1: Academic stress positively predicts employment anxiety.
H2: Academic stress negatively predicts psychological resilience.
H3: Psychological resilience negatively predicts employment anxiety and mediates the relationship between academic stress and employment anxiety.

**Figure 1:**
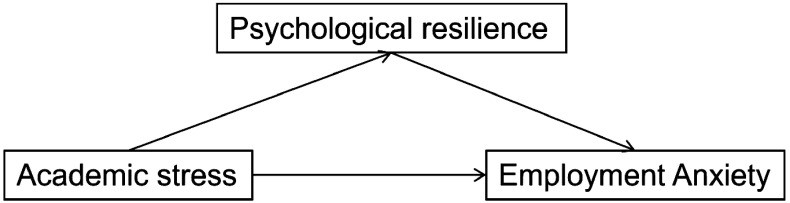
The concept model.

A conceptual model illustrating these hypotheses is presented in **Figure 1**.

This study aims to clarify how academic stress contributes to employment anxiety through the mediating role of psychological resilience. The findings will inform student support strategies and contribute to a better theoretical understanding of resource dynamics in academic and career development based on COR theory.

## 2 Methods

### 2.1 Research design

This study employed a cross-sectional quantitative survey design to examine the mediating role of psychological resilience in the relationship between academic stress and employment anxiety among college students.

### 2.2 Study area

Data were collected from full-time undergraduate students enrolled at three Chinese universities: Zhejiang Gongshang University, Heilongjiang University of Chinese Medicine, and Changchun University of Chinese Medicine. These institutions represent diverse regional and disciplinary contexts in eastern and northeastern China.

### 2.3 Participants

Stratified cluster random sampling method was employed to recruit full-time undergraduate students based on their academic majors and grade levels from October to December 2024. Inclusion criteria were: (1) full-time registered undergraduate students; (2) sufficient reading and comprehension ability to complete an online questionnaire independently; (3) informed and voluntary participation. Exclusion criteria were: (1) a prior diagnosis of severe mental disorders or neurological diseases; (2) current psychological treatment or psychiatric medication use; (3) failure to provide valid informed consent.

According to the sample size estimation principle for mediation analysis, G*Power 3.1 software was used to calculate the minimum required sample size. Assuming a medium effect size (f² = 0.15), *α* = 0.05, power (1–*β*) = 0.95, and five predictors, the minimum required sample size was 138. To improve the model’s stability and account for a potential invalid response rate of 10–20%, the final target sample size was expanded to 1,200. A total of 1,200 questionnaires were distributed, and 1,124 valid responses were collected, resulting in a valid response rate of 93.7%.

### 2.4 Instruments

(1) Demographic Questionnaire: This instrument collected basic information, including gender, grade, academic discipline, only-child status, residence, student leadership experience, internship experience, and monthly household income.

(2) Academic Stress Inventory (ASI): Developed by Lin and Chen (34) the ASI consists of 34 items across seven dimensions: teacher-related stress, outcome stress, exam stress, group study stress, peer pressure, time management stress, and self-imposed stress. Items are rated on a 5-point Likert scale (1= strongly disagree, 5= strongly agree), with higher scores indicating greater perceived academic stress (eg. item *I worry about the consequences of failing an exam.*). This scale has been widely used and validated among university students (35). The Cronbach’s α for the total scale was 0.942. Confirmatory factor analysis (CFA) supported a seven-factor structure with acceptable model fit: χ²/df = 2.74, CFI = 0.938, TLI = 0.926, RMSEA = 0.056, SRMR = 0.045.

(3) Connor-Davidson Resilience Scale-Short Form (CD-RISC-10): Originally developed by Campbell-Sills and Stein (36), the Chinese version was introduced and validated by Lü Zhe et al. for use among Chinese university populations (37). The scale includes 10 items rated on a 5-point scale (0–4), with higher scores indicating greater resilience (eg. item *When things change, I can adapt*). The CD-RISC-10 was selected due to its validated structure in Chinese student samples and its efficiency in measuring resilience without overburdening participants in large-scale psychological surveys. Previous studies have demonstrated that the short form maintains robust construct validity and internal consistency comparable to the full scale (38, 39). In this study, the Cronbach’s α was 0.956. CFA confirmed a unidimensional structure with excellent fit: χ²/df = 1.97, CFI = 0.965, TLI = 0.954, RMSEA = 0.043, SRMR = 0.038.

(4) College Graduates’ Employment Anxiety Questionnaire: Based on the structure proposed by Zhang Yuzhu et al., this 26-item scale assesses four dimensions: job competition pressure, lack of employment support, low self-confidence, and concern about job prospects (40). Items are rated on a 5-point Likert scale, with higher scores reflecting higher levels of employment anxiety (eg. item *I was afraid that my career aspirations would not be realized*). In this study, the Cronbach’s α for the scale was 0.930. CFA indicated a four-factor structure with good model fit: χ²/df = 2.85, CFI = 0.927, TLI = 0.912, RMSEA = 0.059, SRMR = 0.049.

### 2.5 Quality control

The survey was administered online using the “Questionnaire Star.” platform. All participants accessed the questionnaire via a uniform link provided by trained investigators, with restrictions ensuring that each device could submit only once. To enhance data quality, the system set a minimum completion time of 4 minutes and embedded logic reversal and jump-check questions to detect random or invalid responses. Responses with incomplete data, abnormally short completion times, or inconsistent answer patterns were excluded to ensure the validity and reliability of the dataset.

### 2.6 Ethical considerations

This study was approved by the Ethics Committee of the Third Affiliated Hospital of Changchun University of Chinese Medicine (CZDSFYLL2024XS-112). All participants were required to read an electronic informed consent form prior to participation and proceeded to the questionnaire only upon agreement. The entire research process adhered to the ethical principles of voluntary participation, confidentiality, and the right to withdraw at any time. Given the nature of online psychological data collection, particular attention was paid to digital privacy protection, and data were anonymized prior to analysis. These practices align with recent recommendations on the ethical handling and open sharing of psychiatric and behavioral data in digital environments (41).

### 2.7 Statistical analysis

All statistical analyses were performed using IBM SPSS Statistics 26.0. For continuous variables, data conforming to normal distribution were presented as mean ± standard deviation, and categorical variables were described using frequencies and percentages. Pearson correlation analysis was conducted to examine the relationships among key variables. Mediation effects were tested using the Bootstrap method via Hayes’ PROCESS V3.5 macro (Model 4), with 5,000 resamples to generate a 95% bias-corrected confidence interval. Mediating effect was considered significant if the confidence interval did not contain zero. Two-tailed *p*-value less than 0.05 was considered statistically significant.

## 3 Results

### 3.1 Common method deviation test

Before explaining the results of regression and mediation analysis, key statistical hypotheses were evaluated. All variables follow a normal distribution. Also, Harman single factor test was used to test for common method bias in the collected data. The exploratory factor analysis resulted without rotation showed that a total of 10 factors with eigenvalues greater than 1 were extracted, and the variance explanation rate of the largest factor was 25.49%, which was lower than the evaluation standard of 40% common method deviation. Therefore, it can be considered that there is no serious common method deviation in this study.

### 3.2 Demographic characteristics

A total of 1,124 valid questionnaires were collected. Among the participants, 47.86% were male (n = 538) and 52.14% were female (n = 586). Grade one, two, three, and four students accounted for 21.97%, 26.25%, 27.58%, and 24.20% of the sample. Regarding academic disciplines, 35.05% majored in liberal arts, 34.43% in engineering, and 30.52% in medicine. Urban and rural household registrations were reported by 43.86% and 56.14% of students, respectively. Monthly household income was reported as above 10000 yuan by 25.18% of students, 5001~10000 yuan by 42.08%, and below 5000 yuan by 32.74%. Details were presented in **Table 1**.

**Table 1:** Demographic differences in students employment anxiety (n = 1110).

Variable	n (%)	Employment anxiety, Mean ± SD	*F/t*	*p*
Grade			12.430	<0.001
One	247 (21.97)	82.78 ± 13.30		
Two	295 (26.25)	84.53 ± 13.14		
Three	310 (27.58)	86.83 ± 10.33		
Four	272 (24.20)	89.43 ± 16.21		
Gender			7.530	<0.001
Male	538 (47.86)	82.88 ± 12.53		
Female	586 (52.14)	88.79 ± 13.77		
Residence			0.771	0.441
Urban	493 (43.86)	85.61 ± 13.86		
Rural	631 (56.14)	86.24 ± 13.24		
Discipline			1.604	0.202
Arts	394 (35.05)	86.91 ± 13.07		
Engineering	387 (34.43)	85.70 ± 12.95		
Medicine	343 (30.52)	85.19 ± 14.56		
Only child			0.714	0.475
No	323 (28.74)	86.15 ± 13.47		
Yes	801 (71.26)	85.51 ± 13.63		
Student leader			0.218	0.827
No	734 (65.30)	86.03 ± 13.46		
Yes	390 (34.70)	85.84 ± 13.62		
Internship experience			7.289	<0.001
No	618 (54.98)	88.56 ± 13.30		
Yes	506 (45.02)	82.79 ± 13.10		
Monthly income(yuan)			14.931	<0.001
≤5000	368 (32.74)	88.73 ± 14.61		
5001~10000	473 (42.08)	85.56 ± 13.26		
>10000	283 (25.18)	83.04 ± 11.66		

SD, Standard Deviation.

### 3.3 Univariate analysis of employment anxiety across demographic variables

Independent sample t-tests and one-way ANOVAs were conducted to examine differences in employment anxiety across demographic groups. Independent samples t-test indicated that female students reported significantly higher levels of employment anxiety than male students (*t* = 4.32, *p* < 0.001). In terms of grade level, freshmen exhibited significantly higher anxiety than seniors (*F*= 3.94, *p* = 0.009). Students from low-income households also showed elevated anxiety scores compared to those from higher-income backgrounds (*F* = 5.21, *p* = 0.006). No statistically significant differences were found in relation to residence or academic discipline (*p* > 0.05). Results were shown in **Table 1**.

### 3.4 Correlation analysis

The mean scores for perceived Academic stress, psychological resilience, and employment anxiety were 85.96 ± 13.51, 87.94 ± 11.05, and 27.02 ± 6.54, respectively (**Table 2**). Pearson correlation analysis (**Table 2**) revealed that perceived academic stress was negatively correlated with psychological resilience (*r*=-0.349, *p*<0.001) and positively correlated with employment anxiety (*r*=0.428, *p*<0.001). Psychological resilience was also negatively correlated with employment anxiety (*r*=-0.389, *p*<0.001).

**Table 2:** Description statistics and correlation analysis of each variable.

Variables	M ± SD	1	2	3
1.Employment Anxiety	85.96 ± 13.51	1		
2.Perceived Academic stress	87.94 ± 11.05	0.428**	1	
3.Psychological Resilience	27.02 ± 6.54	-0.349**	-0.389**	1

^*^ *p*<0.05, ^**^ *p*<0.01. SD, Standard Deviation.

### 3.5 Mediation analysis

The PROCESS macro Model 4 was used to assess the mediating role of psychological resilience in the relationship between Academic stress and employment anxiety. In the first step, perceived Academic stress significantly predicted employment anxiety (*B*=0.523, *p*<0.001). In the second step, perceived Academic stress significantly predicted psychological resilience (*B*=-0.230, *p*<0.001). In the third step, when both perceived Academic stress and psychological resilience were included as predictors, perceived Academic stress (*B*=0.421, *p*<0.001) and psychological resilience (*B*=-0.444, *p*<0.001) significantly predicted employment anxiety (**Table 3**). The bootstrap method further confirmed the significance of the mediation effect (**Table 4**). The bootstrap analysis revealed that the indirect effect accounted for 19.50% of the total effect (*B*=0.102, 95%CI:0.073 to 0.135), suggesting that a substantial portion of the impact of perceived Academic stress on students’ employment anxiety was mediated through the psychological resilience.

**Table 3:** Summary of hierarchical regression analyses predicting employment anxiety.

Regression equation		Overall fit coefficient			Regression coefficient			
Outcome variables	Predictor variables	*R*	*R^2^*	*F*	*β*	*B*	*SE*	*t*
Employment Anxiety	Perceived Academic stress	0.428	0.183	251.259^**^	0.428	0.523	0.033	15.851^**^
Psychological Resilience	Perceived Academic stress	0.389	0.151	200.014^**^	-0.389	-0.230	0.016	-14.143^**^
Employment Anxiety	Perceived Academic stress	0.471	0.222	159.981^**^	0.344	0.421	0.035	12.040^**^
	Psychological Resilience				-0.215	-0.444	0.059	-7.504^**^

*p<0.05, **p<0.01. CI, Confidence Interval; SE, Standard Error; *β*, Standardized Regression Coefficient; *B*, Unstandardized Regression Coefficient; *R*², Coefficient of Determination.

**Table 4:** Direct and indirect effects of perceived academic stress on employment anxiety.

Path	*B*	*SE*	95%CI	Ratio of effect values
Total effect	0.523	0.033	[0.458,0.588]	
Direct effect	0.421	0.035	[0.352,0.489]	80.50%
Indirect effect	0.102	0.016	[0.073,0.135]	19.50%

### 3.6 Sensitivity analysis

To test the robustness of the mediation model, covariates were selected based on both their statistical significance in univariate analysis and their theoretical relevance as supported by previous literature. Specifically, gender, academic grade, internship experience, and monthly family income were included as control variables. After adjusting for these covariates, the mediation path remained significant, with an indirect effect of 0.092 (95% CI [0.065, 0.121]), accounting for 19.66% of the total effect. The mediation pattern remained stable, indicating strong model robustness. Detailed results are presented in **Tables 5**, **6**.

**Table 5:** Summary of hierarchical regression analyses predicting employment anxiety.

Regression equation		Overall fit coefficient			Regression coefficient			
Outcome variables	Predictor variables	*R*	*R^2^*	*F*	*β*	*B*	*SE*	*t*
Employment Anxiety	Perceived Academic stress	0.527	0.278	86.096^**^	0.383	0.468	0.031	14.878^**^
	Gender				0.166	4.476	0.692	6.464^**^
	Grade				0.122	1.525	0.320	4.774^**^
	Internship experience				-0.117	-2.082	0.456	-4.562^**^
	Monthly income				-0.177	-4.791	0.693	-6.912^**^
Psychological Resilience	Perceived Academic stress	0.394	0.155	41.007^**^	-0.381	-0.226	0.017	-13.707^**^
	Gender				-0.047	-0.616	0.362	-1.699
	Grade				0.006	0.035	0.167	0.210
	Internship experience				0.032	0.272	0.239	1.139
	Monthly income				0.020	0.268	0.363	0.739
Employment Anxiety	Perceived Academic stress	0.558	0.311	83.986^**^	0.308	0.376	0.033	11.315^**^
	Psychological Resilience				-0.197	-0.407	0.059	-7.300^**^
	Gender				0.156	4.225	0.678	6.234^**^
	Grade				0.123	1.539	0.312	4.930^**^
	Internship experience				-0.111	-1.971	0.446	-4.416^**^
	Monthly income				-0.173	-4.681	0.678	-6.908^**^

**p<0.01. CI, Confidence Interval; SE, Standard Error; *β*, Standardized Regression Coefficient; *B*, Unstandardized Regression Coefficient; *R*², Coefficient of Determination.

**Table 6:** Direct and indirect effects of perceived academic stress on employment anxiety.

Path	*B*	*SE*	95%CI	Ratio of effect values
Total effect	0.468	0.031	[0.406,0.530]	
Direct effect	0.376	0.033	[0.311,0.441]	80.34%
Indirect effect	0.092	0.014	[0.065,0.121]	19.66%

## 4 Discussion

This study examined the relationship between academic stress, psychological resilience, and employment anxiety among Chinese university students. The results confirmed that academic stress significantly predicted employment anxiety, and that psychological resilience partially mediated this relationship. These findings support the Conservation of Resources theory and extend prior research by clarifying the underlying mechanism.

Consistent with recent post-pandemic findings, a generally high level of employment anxiety was observed, particularly among female students, lower-year students, and those from lower-income households. Students without internship experience also reported elevated anxiety levels. These patterns suggest that demographic and experiential factors significantly influence students’ career-related stress (32, 42). Female students may face greater societal and familial expectations, while junior students might experience anxiety due to insufficient career preparation (3, 43). Lower-income students may feel greater urgency to secure employment, and lack of internship exposure may heighten uncertainty (44). Furthermore, students without internship experience demonstrated significantly higher anxiety, suggesting that practical exposure to the labor market may help reduce employment-related worries by increasing confidence and familiarity with workplace expectations (45). These insights highlight the need to tailor interventions based on subgroup characteristics, especially in mental health and career guidance programs.

The results extend prior research by demonstrating that academic stress not only exerts a direct influence on employment anxiety but also operates indirectly through reduced resilience. Academic stress, previously associated with depression (46), burnout (47), and emotional exhaustion (8), is now shown to influence anticipatory anxiety about the job market. In this regard, employment anxiety represents a specific and future-oriented psychological outcome that bridges academic and career domains. These findings are particularly relevant in the wake of COVID-19, which has amplified educational disruption and economic instability, compounding young people’s uncertainty about the future (48, 49).

Psychological resilience emerged as a significant mediator in the relationship between academic stress and employment anxiety. Students with higher resilience levels were less likely to experience severe employment anxiety despite high academic stress. This finding aligns with prior studies demonstrating that resilience enhances emotional regulation, buffers the effects of academic and social pressures, and promotes mental health stability among university populations (50, 51). According to the COR theory, individuals strive to obtain, retain, and protect their valued resources. Stress occurs when there is a threat of resource loss, actual resource loss, or lack of resource gain following significant resource investment (52). In the academic context, psychological resilience functions as a vital internal resource that enables students to cope with stressors effectively. It serves as a buffer against the depletion of emotional and psychological resources, thereby mitigating the impact of academic stress on employment anxiety (53). Empirical studies support this theoretical framework. For instance, a study highlighted that coping tendency partially mediates the relationship between psychological resilience and health problems, emphasizing the role of adaptive coping strategies in resource conservation (54).

These findings have practical implications for mental health and career support services in universities. Institutions should prioritize resilience-building programs, especially for early-year students and those without practical work exposure. Interventions such as mindfulness-based stress reduction, strength-based coaching, and resilience training could help students develop adaptive coping strategies. Additionally, integrating resilience assessment into academic advising may allow for more targeted support. As employment anxiety is a complex outcome influenced by both academic and career readiness, dual-focused interventions may be most effective in promoting psychological well-being and post-graduation success.

Finally, the generalizability of these findings is bounded by cultural and methodological factors. The data were collected exclusively online, possibly introducing selection bias toward students with better digital access. Moreover, all variables were measured through self-reported questionnaires, raising concerns about social desirability and common method variance, although reverse-scored items were included. As the sample was drawn from Chinese universities, the findings should be interpreted within the cultural and institutional context of China’s higher education system. Cultural norms around academic success, mental health, and job expectations may limit the generalizability of results. Differences in psychological and career support across countries could also affect coping responses. Future cross-cultural and longitudinal studies are needed to test the model’s applicability and examine the development of resilience over time.

## 5 Conclusion

This study examined the relationship between academic stress and employment anxiety among Chinese university students, with a specific focus on the mediating role of psychological resilience. The results confirmed that academic stress significantly increased levels of employment anxiety, both directly and indirectly through its negative impact on psychological resilience. These findings support the application of the Conservation of Resources theory in explaining students’ psychological reactions to academic and career pressures. By identifying resilience as a key psychological resource that mitigates stress-related anxiety, the study highlights the importance of protective factors in maintaining student mental health during the transition to the workforce. The research contributes to a deeper understanding of how academic demands translate into future-oriented psychological distress and emphasizes the value of resilience-enhancing interventions. These insights may inform the design of more effective psychological support and career development programs within university settings.

## Data Availability

The original contributions presented in the study are included in the article/**Supplementary Material**. Further inquiries can be directed to the corresponding authors.
